# Global inventory and audit of road injury data systems: a descriptive study using the Road Injury Chain of Survival Framework

**DOI:** 10.1186/s13049-026-01633-1

**Published:** 2026-05-27

**Authors:** Tim Nutbeam, Emily Foote, Lauren R. Rodgers, Rob Fenwick, Willem Stassen

**Affiliations:** 1 IMPACT: Centre for Post-Collision Research, Innovation and Translation, Devon Air Ambulance, Exeter, UK; 2 University of Plymouth, Plymouth, UK; 3 University Hospitals Plymouth NHS Trust, Plymouth, UK; 4 Betsi Cadwaladr University Health Board, Wrexham Maelor Hospital, Croesnewedd Road, Wrexham, UK; 5 Wrexham University, Wrexham, UK; 6 Division of Emergency Medicine, Department of Family, Community and Emergency Care, University of Cape Town, Cape Town, South Africa

**Keywords:** Road traffic injury, Injury surveillance, Trauma registries, Emergency medical services, Health system mapping

## Abstract

**Background:**

Road injury causes an estimated 1.19 million deaths annually and tens of millions of non-fatal injuries worldwide. Despite this burden, post-collision data remain fragmented across police, transport, and health sectors, impeding system-level improvement. The recently proposed Road Injury Chain of Survival framework provides a structure for mapping data needs across five sequential links: Early Recognition, Early Rescue, Early Initial Care, Early Transport, and Early Hospital Care & Rehabilitation. This study aims to identify and describe national and major regional road injury datasets worldwide and to assess their coverage against a novel RICS-derived sub-indicator framework.

**Methods:**

A structured, descriptive global audit was undertaken to identify and characterise national and major regional road injury datasets. Searches included government and institutional portals, international repositories, and peer-reviewed literature. Eligible datasets provided national or nationally aggregated information on road traffic collisions, injuries, or trauma care. Each dataset was assessed against 20 sub-indicators (four per chain link) using a three-point coding system: 1 (present), 0.5 (partial/proxy), 0 (absent), or U (unverified).

**Results:**

A total of 349 national and major regional road injury data systems were identified worldwide, representing 357 datasets after disaggregation of linked systems. Police crash databases were the most common data source across all income groups and were near-universal in low-income countries. Mortality-focused datasets predominated in lower-income countries while higher-income countries demonstrated greater diversity, including emergency medical services, fire and rescue, and trauma registry data. Formal cross-sector data linkage was rare, and most datasets were available only in aggregated form. Mapping to the Road Injury Chain of Survival showed that data were most frequently available for Early Recognition, with substantially less coverage for the remaining links in the chain.

**Conclusions:**

This global audit of road injury datasets reveals extensive fragmentation across the post-collision care continuum. Few systems link crash, rescue, prehospital, and hospital data, severely limiting opportunities for quality improvement and outcome evaluation. Development of harmonised, interoperable datasets aligned with the Road Injury Chain of Survival is essential to enable benchmarking, guide investment, and improve outcomes from road injury worldwide.

**Pre-registered:**

Open Science Framework: 10.17605/OSF.IO/VXE6S

**Supplementary Information:**

The online version contains supplementary material available at 10.1186/s13049-026-01633-1.

## Background

 Road injury remains a leading cause of death and disability worldwide, accounting for an estimated 1.19 million deaths annually and tens of millions of non-fatal injuries with substantial social and economic consequences [1]. Meaningful improvement in post-collision outcomes requires not only effective interventions but also the systematic collection, linkage, and use of data across the entire care pathway. The “chain of survival” concept has transformed outcomes in out-of-hospital cardiac arrest (OHCA) by standardising what is measured, enabling benchmarking between systems, and driving quality improvement at scale [2]. Population-based studies demonstrate that strengthening successive links in the OHCA chain; community response, dispatch, basic and advanced life support, and post-resuscitation care has been associated with sustained gains in survival over time [2].

Central to these gains has been the development and spread of high-quality registries based on harmonised reporting templates (the Utstein framework) that enable comparable data capture, transparent performance metrics, and targeted system change. The Cardiac Arrest Registry to Enhance Survival (CARES) and pan-European initiatives (e.g. EuReCa), alongside trauma registry data such as that generated by the Trauma Audit and Research Network (TARN), illustrate how consistent data structures facilitate benchmarking, reveal inter-system variation, and help focus improvement efforts where the potential impact is greatest [3–5]. Recent updates to the Utstein templates and Global Resuscitation Alliance recommendations further emphasise registry-driven quality improvement as the foundation for population-level survival gains [6].

A comparable, end-to-end framework has been proposed for road injury; the Road Injury Chain of Survival (RICS); spanning five sequential links: Early Recognition, Early Rescue, Early Initial Care, Early Transport, and Early Hospital Care & Rehabilitation [7]. The RICS adapts the logic of the OHCA chain to the distinct priorities of post-collision care and offers a common language for research, service design, and evaluation. Just as in OHCA, the utility of the framework extends beyond a conceptual model: it can guide what data to collect at each link, how to align definitions across agencies (police, fire and rescue, emergency medical services (EMS), trauma systems), and where to target improvement and investment.

Unlike cardiac arrest, the data landscape for road injury is fragmented across multiple custodians and frequently siloed between transport, law enforcement, and health sectors [8]. National police crash statistics are near-universal but typically lack prehospital clinical content; fire and rescue datasets capture rescue activities but are not commonly linked to clinical outcomes; trauma registries provide rich in-hospital data but may have incomplete prehospital fields or limited national coverage. Without a consolidated view of what exists and where the gaps are it is difficult to design linkage strategies, set minimum data standards, or prioritise feasible, high-yield improvements.

This study is designed to address that gap. We conducted a structured, descriptive global audit and systems mapping study of national and major regional road injury datasets, assessing them against a novel sub-indicator framework derived from the RICS. Specifically, our objectives were to: (1) identify and catalogue datasets across countries and custodians; (2) evaluate the extent to which key sub-indicators are present, partially represented, or absent at each RICS link; and (3) explore opportunities for future standardisation, data linkage, and system improvement.

By taking this approach, we seek to provide the same type of analytic “lens” that cardiac arrest registries have provided for resuscitation systems, clarity on what is currently measured, where it is measured, and how this information could be harnessed to improve outcomes following road injury.

## Methods

### Study design

We conducted a structured, descriptive global audit and systems mapping study to identify and characterise national and major regional road injury datasets worldwide. The primary aim was to assess the extent to which these datasets capture information relevant to each stage of the RICS. The study was designed as a point-in-time infrastructure inventory, using predefined eligibility criteria, a multi-source discovery strategy, and a standardised sub-indicator framework to enable consistent classification and comparison of heterogeneous data systems. The study protocol and analytic approach were pre-registered with the Open Science Framework [9].

### Identification of countries

The audit sought to include all sovereign states, observer states, and dependent territories recognised by the United Nations, the World Health Organization (WHO), or the World Bank classification systems. Overseas departments and territories were combined with their parent country to avoid duplication. Federal states (for example, the United States, Brazil, and India) were treated as national entities. Sub-national or state-level registries within these countries were excluded unless their data were explicitly aggregated and reported at national level.

### Identification of datasets

#### Government and institutional sources

We conducted a structured desk-based audit of publicly available online resources, including national government and institutional portals (for example, transport ministries, health ministries, national statistics offices, and fire and rescue authorities). In parallel, structured web searches were undertaken using Google, applying predefined keyword families combined with country names (for example, “road traffic injury statistics [country name]”, “national trauma registry [country name]”, and “EMS incident data [country name]”).

This discovery strategy was designed to identify operational data systems that are not routinely indexed in bibliographic databases but are accessible through official websites, statistical releases, and government or institutional reports.

#### Scientific literature

We searched PubMed and Google Scholar for peer-reviewed publications describing national or nationally aggregated road traffic injury datasets, trauma registries, or large-scale crash databases. Searches were limited to publications from January 2023 to September 2025 in order to identify contemporaneous data systems and recently established or updated registries.

The search strategy combined controlled vocabulary (MeSH) and free-text terms, including:

“road traffic injuries” OR “road injury” OR “motor vehicle collisions”

AND “registry” OR “database” OR “data system” OR “surveillance” OR “statistics”.

AND “national” OR “population-based.”

Reference lists of relevant articles were also screened (“snowballing”) to identify additional eligible datasets.

#### International compendia

We reviewed international repositories including the World Health Organization’s *Global Status Report on Road Safety* and the OECD/International Road Traffic Accident Database (IRTAD) [10]. Candidate datasets identified through government portals, institutional sources, scientific literature, and international compendia were independently reviewed against the predefined inclusion and exclusion criteria. Disagreements regarding eligibility or classification were resolved by consensus, with reference to publicly available custodian documentation and registry descriptions.

##### Language considerations

Only publications and datasets available in English were considered directly. Where materials were published in other languages but translatable via Google Translate, they were included if the translation provided sufficient clarity to identify dataset scope and content.

##### Eligibility assessment

All identified sources were collated, screened for duplicates, and assessed against predefined inclusion and exclusion criteria.

##### Inclusion and exclusion criteria

The inclusion and exclusion criteria were developed a priori and applied consistently across countries.

Datasets were eligible for inclusion if they met one or more of the following criteria:

- National or nationally aggregated datasets relating to road traffic collisions, injuries, or trauma care.
- Major regional systems that either operate at or near national scale, or that provide exceptional depth and breadth of data across multiple domains (for example, Road Accident In-Depth Studies (RAIDS) in the United Kingdom and the Swedish Traffic Accident Data Acquisition system (STRADA)).
- Trauma registries with explicit or identifiable coverage of road traffic injury patients.
- Mortality databases where dedicated tables or modules relating to road traffic injury are available.
- Emergency medical services (EMS) and Fire & Rescue incident reporting systems that include road traffic collisions as a defined incident category.

Datasets were excluded if they met any of the following criteria.

- Sub-national registries within federal systems, unless data were explicitly aggregated and reported at national level.
- Research datasets with limited geographical scope (for example, single-hospital or regional pilot studies).
- Databases without publicly available information or verifiable descriptions of dataset scope and content.

### Development of the sub-indicator framework

To evaluate the relevance of each dataset to the RICS, we developed a novel sub-indicator framework.

The sub-indicators were generated through a staged development process. We first undertook a review of existing measurement frameworks, including the World Health Organization’s road safety monitoring indicators, the Utstein templates for trauma, and established trauma registry data dictionaries. From this foundation, we generated a preliminary list of candidate indicators that could reasonably be expected to describe activity at each RICS link. This list was then iteratively refined through discussion among the study authors, with particular emphasis on ensuring that sub-indicators were both specific (relating to a discrete measurable variable, such as “extrication duration” or “prehospital vital signs recorded”) and broadly applicable across different health systems and data environments.

To test feasibility and construct validity, the draft framework was applied to a set of exemplar datasets from the United Kingdom, including STATS19 police crash data, the RAIDS dataset, and the National Major Trauma Registry (formerly TARN). This pilot exercise confirmed that the sub-indicators could discriminate between datasets with differing levels of clinical and operational detail and informed the development of a graded coding scheme. The final framework consisted of 20 sub-indicators, with four sub-indicators mapped to each RICS link.

Each dataset was then assessed against the sub-indicators using a four-category coding scheme: 1 if the variable was explicitly represented in the dataset; 0.5 if only partial or proxy data were present (for example, “fatal/serious/slight injury” as a proxy for in-hospital outcome); 0 if the variable was absent; and U if the presence of the variable could not be verified from publicly available sources. For classification purposes, datasets were defined as open if available for download or query by any user without a formal application or approval process. Datasets accessible only under a data use agreement, institutional data sharing agreement, or equivalent formal access mechanism were classified as restricted, irrespective of whether such access was routinely granted. This approach enabled consistent comparison across heterogeneous datasets while retaining transparency regarding uncertainty and data observability Table 1.

**Table 1:** Sub-indicator framework

Link	Sub-indicator code	Sub-indicator description
Link 1 – Early Recognition	1.1	Date/time/location of crash
	1.2	Notification to emergency services (call receipt, eCall trigger)
	1.3	Bystander detection or reporting captured
	1.4	Survival status at scene (dead/alive on discovery)
Link 2 – Early Rescue	2.1	Entrapment status
	2.2	Fire/rescue attendance
	2.3	Extrication start/finish time or duration
	2.4	Number of persons released/rescued
Link 3 – Early Initial Care	3.1	Prehospital physiology (vital signs recorded)
	3.2	Prehospital interventions (airway, Tranexamic acid (TXA), Intravenous (IV) analgesia)
	3.3	Bystander care or first aid documented
	3.4	EMS provider type/level (paramedic, physician, etc.)
Link 4 – Early Transport	4.1	Mode of transport (road, air, other)
	4.2	Time from scene to hospital
	4.3	Receiving hospital / destination coded
	4.4	Secondary transfer to another facility
Link 5 – Early Hospital Care & Rehabilitation	5.1	Hospital admission (yes/no, ward/Intensive Care Unit (ITU))
	5.2	In-hospital interventions (surgery, transfusion, imaging)
	5.3	In-hospital outcomes (mortality, discharge, length of stay)
	5.4	Rehabilitation referral or follow-up

### Coding of datasets

Datasets were coded independently by pairs of reviewers with expertise in trauma, emergency medicine, and road injury epidemiology, working in tandem and cross-checking each other’s classifications. Coding decisions were informed by triangulation of multiple publicly available sources, including official data dictionaries, registry manuals, custodian documentation, and peer-reviewed publications. Sub-indicators were coded as “U” (unverified) only where the presence or absence of a variable could not be confirmed from publicly accessible information, despite this triangulation. In these cases, “U” denoted limited external observability rather than a confirmed absence of the variable within the underlying dataset.

Discrepancies between reviewers were resolved through discussion and consensus, with reference to the predefined coding framework.

### Relationship to the utstein road injury project

This audit was conducted as foundational work for the Utstein Road Injury Project, a planned international consensus initiative to develop standardised reporting for road injury. The sub-indicator framework presented here is intended to characterise the current global data landscape and inform subsequent consensus development, rather than to prescribe a final dataset specification.

## Results

When assessed against the Road Injury Chain of Survival, most identified datasets mapped to a single link in the chain. Coverage was most frequently observed for Early Recognition, commonly through police crash databases recording crash timing, location, and fatality status. In contrast, sub-indicators relating to Early Rescue, Early Initial Care, and Early Transport were infrequently identifiable and were limited to a small number of emergency medical services, fire and rescue, and trauma registry datasets, primarily in high-income countries. Datasets providing observable coverage across multiple links of the chain were uncommon. Throughout, findings reflect what was externally observable from publicly available documentation; operational datasets may exist in some settings where public reporting is limited or restricted, and this constraint should be borne in mind when interpreting the results that follow.

**Fig. 1:**
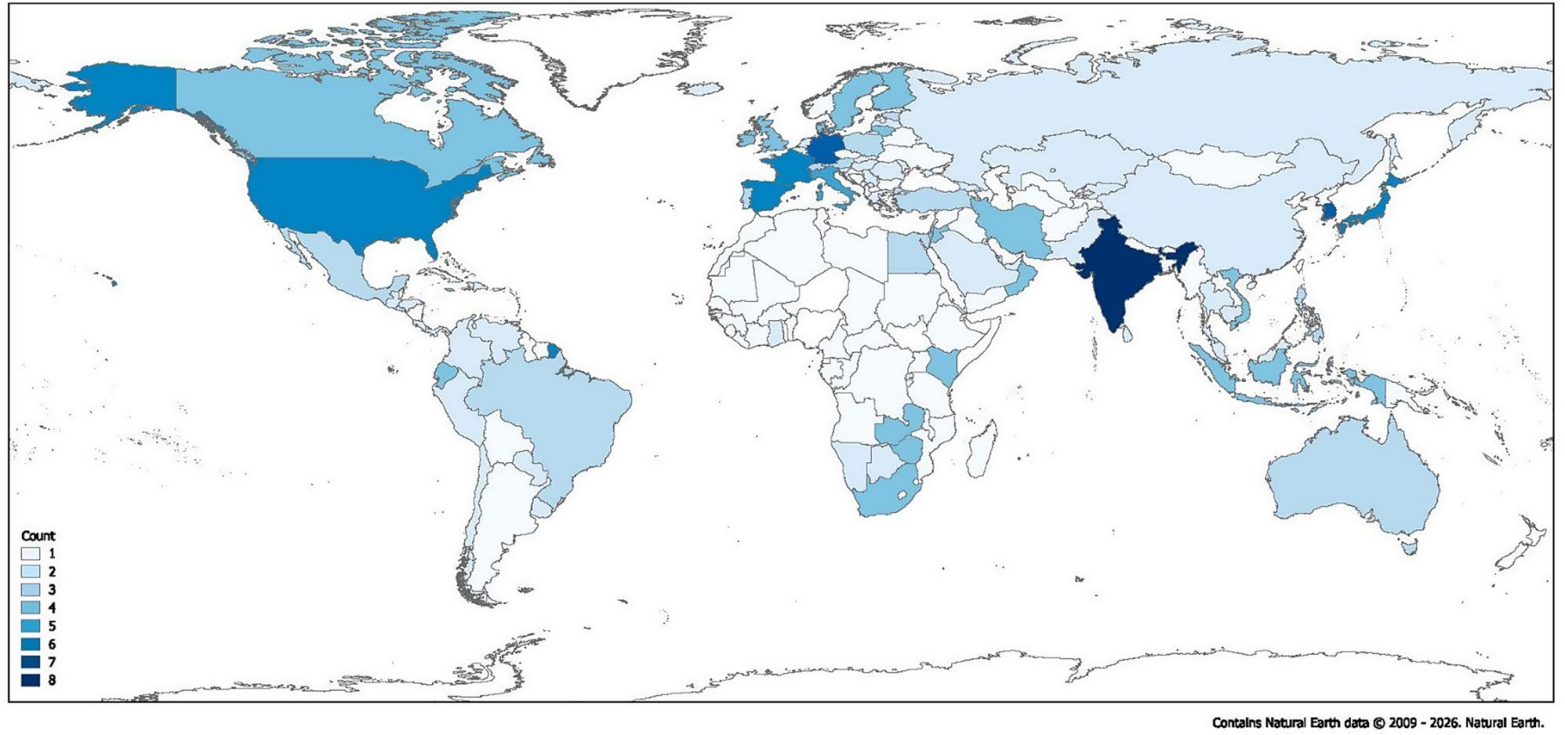
Database count per country

A total of 349 distinct national and major regional road injury data systems were identified globally (Fig. 1, Supplementary Table 1).

For summary analyses by database type, systems that formally linked multiple sectors (for example, EMS and Fire & Rescue) were disaggregated into their component sources in order to allow consistent classification by custodian and dataset category. Using this approach, eight linked systems were represented across the summary tables as their constituent datasets, resulting in a total analytical sample of 357 datasets. Police and crash databases were the dominant data type across all income groups. Dataset diversity increased correspondingly with income: emergency medical services, fire and rescue, trauma registry, and in-depth datasets were rare or absent in low- and lower-middle-income countries but collectively accounted for over a third of datasets in high-income settings. Table 2 summarises the distribution of dataset types by income group.

**Table 2:** Distribution of national and major regional road injury datasets by database type and World Bank income group

Dataset Type	World Bank Income Group				Total
	LIC (*n* %)	LMIC (*n* %)	UMIC (*n* %)	HIC (*n* %)	
Police / Crash Data	23 (95.8)	51 (63.7)	49 (56.3)	62 (37.8)	185 (52.1)
Mortality	1 (4.2)	16 (20.0)	24 (27.6)	40 (24.4)	81 (22.8)
Emergency Medical Services	0	4 (5.0)	5 (5.7)	15 (9.1)	24 (6.8)
Fire & Rescue	0	3 (3.8)	4 (4.6)	13 (7.9)	20 (5.6)
Trauma Registry & Injury Surveillance	0	6 (7.5)	3 (3.4)	20 (12.2)	29 (8.2)
In-Depth & Specialist	0	0	2 (2.3)	14 (8.5)	16 (4.5)
Total	24	80	87	164	355

LIC = Low-income country; LMIC = Lower-middle-income country; UMIC = Upper-middle-income country; HIC = High-income country. Cell shading reflects the proportion of each income group’s datasets represented by that category: darker = higher proportion. Percentages are calculated within each income group column

**Fig. 2:**
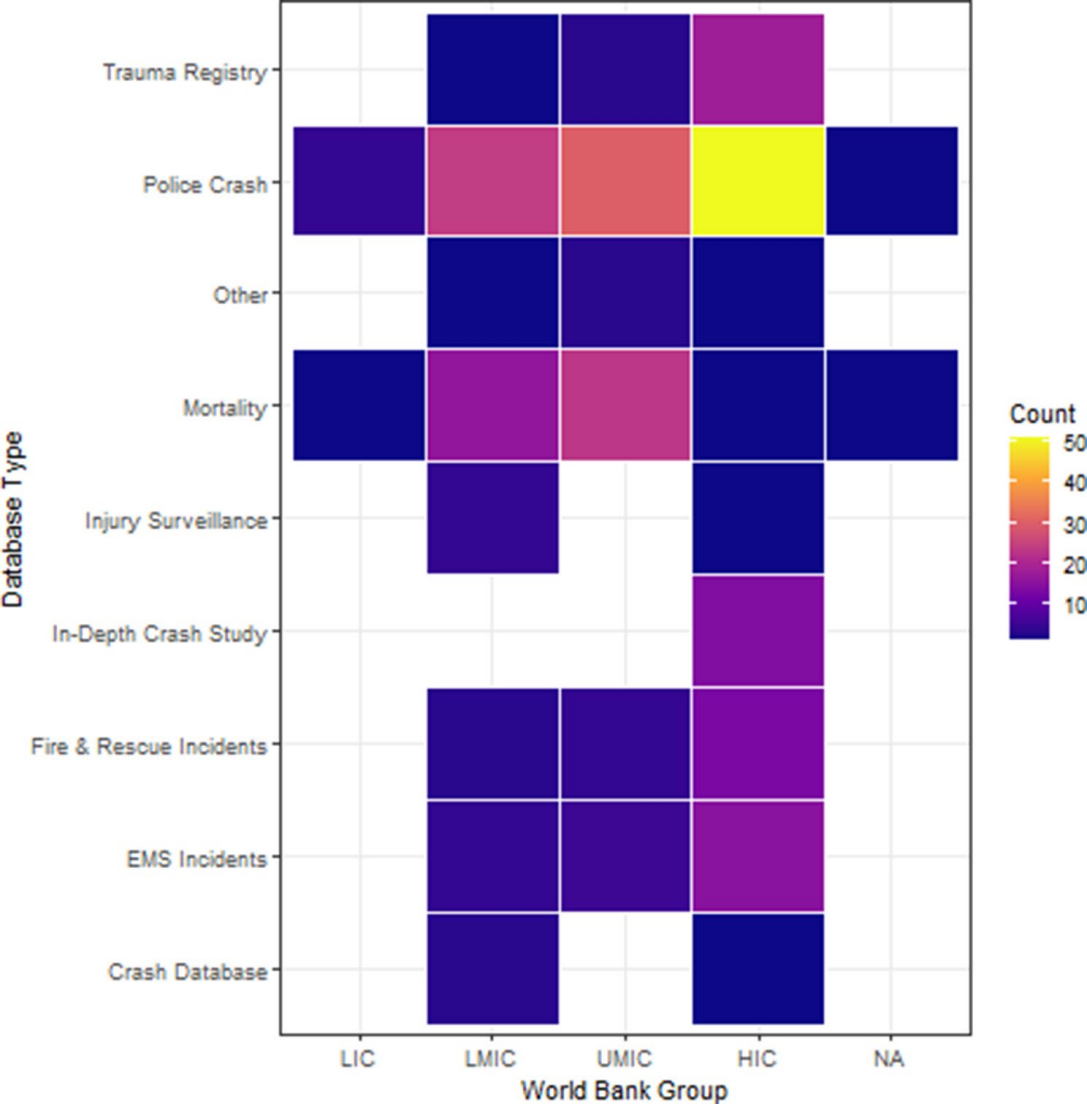
Heat map of database type and world bank income group

The focus of data collection varied across World Bank income groups (Fig. 2). In low-income countries (LICs), the majority of identified datasets related exclusively to police mortality records, accounting for 83.3% (*n* = 20/24) of databases. Mortality-only datasets accounted for 50.0% (*n* = 40/80) in lower-middle-income countries (LMICs), 44.8% (*n* = 39/87) in upper-middle-income countries (UMICs), and 37.9% (*n* = 47/124) in high-income countries (HICs). National mortality databases; held by statistics offices, health ministries, or coronial services and ICD-coded are distinct in custodianship, scope, and data structure from police crash databases that record fatality status as part of crash reporting; the income group-specific ICD coding figures reported are more characteristic of the former category.

Across all income groups, police crash databases were the most frequently identified data source. In low-income countries (LICs), 95.8% (*n* = 23) of datasets were police-based. Police datasets accounted for 60.0% (*n* = 48) of datasets in lower-middle-income countries (LMICs), 56.3% (*n* = 49) in upper-middle-income countries (UMICs), and 37.2% (*n* = 61) in high-income countries (HICs). Publicly available documentation did not consistently permit characterisation of the full content of individual police databases beyond their predominant data type. While some police databases capture road characteristics, vehicle involvement, and injury severity data alongside fatality status, the extent of this additional content varied across countries and could not be uniformly determined from public sources. In higher-income groups, a broader range of dataset types was identified, including health-sector databases, emergency medical services (EMS) datasets, trauma registries, and Fire and Rescue service records. These dataset types functioned as structural proxies for different links in the Road Injury Chain of Survival, with police datasets primarily reflecting Early Recognition and health-sector datasets mapping to later stages of care.

Among datasets that explicitly reported mortality outcomes, the use of ICD-based coding varied by income group. ICD coding was present in 87.5% (*n* = 42/48) of HIC mortality datasets, 69.2% (*n* = 27/39) in UMICs, 47.5% (*n* = 19/40) in LMICs, and 15.0% (*n* = 3/20) in LICs.

Formal linkage between datasets from different sectors was uncommon. Eight linked systems combined EMS and Fire and Rescue data; six were identified in HICs, one in a UMIC, and one in a LIC. Two systems linked police and health data, one in a HIC and one in a LMIC.

Most datasets were available only in aggregated form, accounting for 84.9% (*n* = 303/357) of all identified datasets. Disaggregated, record-level data were therefore available for a minority of systems.

Access to data varied substantially by income group. No datasets from LICs (0%, *n* = 0/24) were available as open data. Open access was observed in 18.8% (*n* = 15/80) of LMIC datasets, 52.9% (*n* = 46/87) of UMIC datasets, and 60.4% (*n* = 99/164) of HIC datasets. Overall, 44.8% (*n* = 160/357) of datasets were classified as open. Of these, four datasets provided open access to disaggregated data, all of which were based in HICs.

In contrast, restricted-access datasets were more likely to provide disaggregated data, with 97.6% (*n* = 41/42) of restricted datasets offering record-level access.

When mapped to the RICS, dataset coverage varied by link. Interpretation was limited by the large proportion of unclassified sub-indicators resulting from restricted access or insufficient publicly available documentation. Data relevant to Early Recognition were most frequently identified and were predominantly derived from police crash databases, typically capturing crash timing, location, and fatality status.

Data relevant to Early Rescue, Early Initial Care, and Early Transport were identified in only a small number of datasets, mainly within EMS, Fire and Rescue, and trauma registry sources, and were largely confined to higher-income countries. Data relevant to Early Hospital Care and Rehabilitation were identified more frequently than for the intermediate links, primarily through hospital-based datasets and national trauma registries, with in-hospital admission and outcome variables reported more commonly than rehabilitation or follow-up data.

## Discussion

Although this study was explicitly framed around the Road Injury Chain of Survival, its central finding is the limited extent to which the chain can be empirically observed using existing national and major regional data systems. When applied as an analytic lens, the RICS primarily exposes the absence, fragmentation, and sectoral silos of post-collision data, rather than a coherent, chain-level picture of care. In most countries, publicly available datasets permit assessment of only isolated segments of the post-collision pathway, most commonly Early Recognition, with little visibility of rescue processes, prehospital care, or transport intervals. This lack of chain-level observability represents a fundamental constraint on system evaluation and improvement, rather than a failure of the framework itself.

This study provides the first global audit of national and major regional road injury data systems mapped against the RICS. The audit demonstrates substantial variation in the availability and public observability of road injury data across the post-collision care pathway, with many systems lacking sufficient publicly available documentation to permit full classification. Very few data systems spanned more than a single RICS link, and almost none provided comprehensive coverage across the continuum of care. Consequently, publicly available sources rarely allow the patient journey to be traced from the scene of the collision through to hospital discharge or rehabilitation.

These findings reflect persistent structural fragmentation, with transport, police, fire and rescue, emergency medical services, and hospitals typically maintaining separate datasets with limited integration. The small number of identifiable linked systems highlights the continuing challenges of connecting crash, rescue, prehospital, and hospital data in a way that supports end-to-end system evaluation and quality improvement.

A key strength of this study is its comprehensive global scope. By applying the RICS framework and a standardised sub-indicator architecture, we were able to benchmark the content of national and major regional road injury data systems across each stage of the post-collision pathway and to identify where critical deficits occur. The use of multiple discovery sources, including government and institutional portals, scientific literature, and international repositories, reduces the likelihood that major operational data systems were overlooked. In addition, the paired-reviewer audit and classification process, together with a transparent coding framework, supports the reproducibility of the approach and its application in future system-mapping and standardisation initiatives.

Several limitations should be acknowledged. This was a descriptive infrastructure audit rather than an evaluation of data quality; we did not assess the completeness, accuracy, or timeliness of the underlying data captured within individual systems. Our reliance on publicly available documentation means that some data systems may be more comprehensive than is externally observable, particularly where access is restricted or documentation is limited. This may have led to under-ascertainment, especially in countries where datasets exist but are not widely reported. Some systems originally established for research purposes may no longer be actively maintained, raising concerns about currency and sustainability.

Our results align with previous work highlighting limitations of police crash databases. Prior research has shown that police records often under-represent the true burden of non-fatal injury, with incomplete capture of certain groups such as pedestrians and cyclists [8]. Studies linking police and hospital data suggest that health-sector records provide a more complete picture of serious injuries, although such linkages are rarely available at national scale [8]. Reviews of trauma registry coverage similarly indicate that relatively few countries maintain national trauma registries, and these are concentrated to high-income settings. Where registries exist, their content and definitions vary considerably, which limits comparability across systems [11]. Our audit extends these observations to a global level, showing that while police crash statistics are near-universal, datasets capturing rescue, prehospital care, transport, and rehabilitation remain uncommon and are rarely integrated.

Given the predominance of police crash datasets and the wide variation in the visibility and accessibility of other data sources, a staged or tiered approach to data specification may offer a pragmatic means of supporting comparability across settings. Such an approach could recognise police-based data as a consistent foundation, with progressive expansion to include rescue, prehospital, transport, and hospital data as system capacity and linkage mechanisms allow.

The World Health Organization’s Global Status Reports on Road Safety note persistent discrepancies between reported fatalities and modelled estimates, and underline the lack of standardised measures for serious injury [1]. Our audit adds to this evidence by demonstrating that the problem is not only under-ascertainment, but also fragmentation across sectors and phases of care. Few countries could be shown, through publicly available sources, to link crash circumstances, rescue data, EMS processes, and hospital outcomes in a way that would allow systematic analysis. Limited data integration has long been identified as a barrier to improving trauma care; our results provide quantitative evidence of the scale of this challenge [11].

The reasons for these gaps are multifactorial. Road crashes have historically been framed as transport and enforcement issues, leading to an emphasis on police crash reports rather than medical outcomes. EMS and hospital sectors often lack mandates or infrastructure for national reporting. Fragmentation across multiple custodians, combined with limited resources(particularly in low- and middle-income countries), further hampers the development of linked datasets. A lack of standardised definitions (for example, of “serious injury” or time-critical intervals) further compounds these difficulties [12].

The implications are substantial. Without linked data, systems cannot identify where along the chain delays occur, which interventions are most effective, or how resources might be better allocated. In essence, it is not possible to improve what is not measured. Quality improvement cycles, such as those that have transformed cardiac arrest outcomes, remain out of reach for most road injury systems without registry infrastructure [4]. It is important to acknowledge that substantial road safety gains in high-income countries, including reductions attributable to speed and alcohol legislation, seat belt and helmet mandates, and the regionalisation of major trauma care, were achieved through population-level policy and system interventions that did not require linked multi-sector datasets. Linked datasets provide something qualitatively different: granular visibility of where within the care pathway delays and failures occur, comparative benchmarking of care processes between systems, and the capacity to evaluate specific clinical and operational interventions at population scale. The eight linked systems identified in this audit demonstrate that cross-sector linkage is achievable across income settings and provide an instructive model for expansion [13].

Our findings underline the urgent need for coordinated effort. First, agreement on a minimum RICS dataset across police, fire/rescue, EMS, hospitals, and rehabilitation is required, with harmonised definitions to ensure comparability [13]. Second, mechanisms for data linkage between sectors should be prioritised, using common identifiers and inter-agency agreements. Third, countries should invest in registry infrastructure, drawing on successful models in cardiac arrest and trauma, where data-driven audit has contributed to improved survival [2]. Finally, international collaboration is essential: just as the Utstein template enabled benchmarking in cardiac arrest, a similar standard for road injury could catalyse system improvement worldwide [7].

Research is needed to identify cost-effective models for establishing RICS-aligned datasets in resource-constrained settings. Mobile data collection platforms, including smartphone-based incident reporting tools and electronic patient report forms integrated with EMS dispatch systems offer a feasible route to capturing structured prehospital data without the infrastructure requirements of traditional registry systems [11]. Governance frameworks that enable multi-agency data sharing; including data use agreements, defined information governance standards, and shared custodianship models, while protecting individual privacy, require further development and formal evaluation [13]. As new systems are developed, rigorous evaluation should examine whether registry implementation translates into improved patient outcomes, building the case for sustained investment. Finally, linked datasets will allow future research to determine which interventions along the chain of survival yield the greatest impact.

## Conclusion

This global audit shows that while police crash data are nearly universal, the vast majority of countries lack linked datasets spanning rescue, prehospital care, transport, and hospital outcomes. Without improved visibility, linkage, and accessibility of data across the post-collision pathway, opportunities for system-level learning and improvement are likely to remain limited. Developing more transparent, interoperable road injury datasets aligned with the Road Injury Chain of Survival would enable robust benchmarking, evaluation, and learning across systems. Differences in data availability and accessibility across income groups further constrain comparative analysis, with disaggregated and linkable datasets most often identifiable in high-income settings. Building this infrastructure will not only enable better science, but also contribute to safer roads, more efficient systems, and improved outcomes for patients.

## Electronic Supplementary Material

Below is the link to the electronic supplementary material.

Supplementary Material 1

## Data Availability

All data generated or analysed during this study are included in this published article or the supplementary file.
